# Chandelier-Assisted Scleral Buckling: A Literature Review

**DOI:** 10.3390/vision7030047

**Published:** 2023-06-28

**Authors:** Lorenzo Governatori, Alessandra Scampoli, Carola Culiersi, Patrizio Bernardinelli, Stefano Maria Picardi, Federica Sarati, Tomaso Caporossi

**Affiliations:** 1 Vitreoretinal Surgery Unit, Fatebenefratelli Isola Tiberina Gemelli Isola Hospital, Catholic University “Sacro Cuore”, 00186 Rome, Italy; alescampoli@gmail.com (A.S.); tomaso.caporossi@gmail.com (T.C.); 2 Ophthalmology Unit, Fondazione Policlinico Universitario A. Gemelli IRCCS, Catholic University “Sacro Cuore”, 00168 Rome, Italy; c.carola@live.it (C.C.); patrizio.bernardinelli@gmail.com (P.B.); 3 Ophthalmology Unit, ASST Melegnano e della Martesana, 20070 Vizzolo Predabissi, Italy; ste.picardi@gmail.com; 4 Eye Clinic, Neuromuscular and Sense Organs Department, University of Florence, 50134 Florence, Italy; federicasarati@gmail.com; 5 Department of Neurosciences, Psychology, Drug Research and Child Health Careggi University Hospital, University of Florence, 50134 Florence, Italy

**Keywords:** retinal detachment, scleral buckling, chandelier, endoilluminator, wide-angle viewing system, 3D visualization system

## Abstract

The treatment of retinal detachment (RD) has seen numerous advancements in the last decades. Scleral buckling (SB) is a surgical procedure introduced in the 1950s that has seen a drastic reduction with the advent of vitrectomy. However, due to the new surgical visualization systems, SB has evolved and continues to be an extremely useful procedure in certain conditions. The presence of different case reports or interventional studies with comparable outcomes, as well as the lack of recent studies with direct comparison, may result in an underestimation of its potential nowadays. The aim of this review is to provide a comprehensive update on chandelier-assisted scleral bucking (CSB), with an overview of the surgical evolution, outcomes, advantages, and complications.

## 1. Introduction

A relatively frequent cause of avoidable blindness is rhegmatogenous retinal detachment (RRD). Its incidence has been estimated to range from 6.3 to 17.9 instances per 100,000 people [1]. Finding and sealing all of the retinal breaks is essential for the effective therapy of retinal detachment (RD). Scleral bucking (SB) or pars plana vitrectomy (PPV) are the two major surgical procedures utilized to accomplish this. Scleral bucking (SB) has been a valuable and effective method since its invention in the 1950s, when Ernst Custodis created a polyviol exoplant to buckle the sclera without draining subretinal fluid, trying to overcome the risks of more dangerous, previous techniques such as full thickness or lamellar scleral resection [2,3]. However, even lamellar resection carried a significant risk of comorbidity; as a result, efforts to duplicate the buckling effect without running the risk of tissue resection were made. These methods included Weve’s reefing operations, scleral out-folding procedures, scleral in-folding procedures, and scleral shortening by shrinkage with diathermy. The primary goal of all of these approaches was the ‘buckling’ effect that was generated, which turned out to be a crucial element in a successful retinal reattachment [4]. Later on, Charles Schepens developed diathermy and a polyethylene circumferential tube to remove subretinal fluid [5]. Harvey Lincoff, probably more than anyone else, made numerous significant and diverse contributions to the development of scleral buckling. He developed new scleral suture needles, introduced silicone sponges as explants, and published the benefits of cryotherapy over diathermy. Further, he stressed the importance of locating the retinal breaks (now known as “Lincoff’s rules”), the significance of buckle orientation for various configurations of retinal break, and the lower comorbidity associated with non-drainage of subretinal fluid [6].

There are numerous different surgical procedures for treating scleral buckling; however, the effectiveness of these procedures depends mostly on a few essential principles. The basic goal is to seal all retinal breaks and maintain closure by preventing any more recruitment of subretinal fluid for as long as is necessary for a retinal adhesion to develop its full strength. The majority of scleral buckling errors are caused by either missing primary breaks or incorrect localization of the buckle, which results in an ineffective seal of the break. The most significant variables that determine whether this surgical approach will be successful are to locate and identify all retinal breaks, for which a complete preoperative and intraoperative examination is required [7]; accurate retinopexy; and finally, appropriate buckle selection and localization to occlude the retinal defect and preserve the retinal pigmented epithelium (RPE) and the neurosensory retina’s apposition.

A complete peritomy should be performed and Tenon’s capsule/conjunctival complex is not disturbed by opening the conjunctiva 2–3 mm posterior to the limbus. This distance from the limbus allows optimal scleral access while minimizing scarring and protecting the limbal stem cell array. The four rectus muscles are hooked and isolated on a silk bridle. At this stage, considerable care is needed to prevent splitting the muscle, slinging an oblique, or removing the muscle capsule, all of which raise the chances of restrictive diplopia after surgery. After inspection, each retinal break must be localized, marked, treated with cryotherapy and finally retinopexy is carefully used to treat them precisely. The next crucial step after choosing the right kind of buckle is precise suture placement, which will define not only where to position the buckle over the break but also the height of the buckle. Subretinal fluid drainage with preceding choroidal diathermy, modification of the buckle height, final fundus evaluation, and conjunctival closure are the last surgical steps. Despite the risk of intraoperative and postoperative complications, such as choroidal/subretinal/retinal bleeding, vitreous/retinal incarceration, retinal perforation and hypotony [8], scleral bucking (SB) still remains the preferred approach for rhegmatogenous retinal detachment (RRD) treatment in phakic young individuals, high myopic eyes with firmly attached hyaloid, absence of proliferative vitreoretinopathy, retinal breaks anterior to the equator, inferior breaks, or retinal dialysis [9,10]. Long-term follow-up studies report a 95% final success rate, while with careful case selection, even a 99% success rate [4]. In classic scleral buckling surgery, an indirect ophthalmoscope is used to see the fundus. Charles Schepens made a significant contribution by creating and popularizing the binocular indirect ophthalmoscope [11]. Indirect ophthalmoscopy produces a picture that is upside down, and using it requires practice. Additionally, patients with a small pupil and posterior capsular opacification may make it difficult to inspect retinal defects. Due to reduced visibility with indirect ophthalmoscopy compared to phakic individuals, scleral buckling may be less preferable in patients with pseudo-phakia or aphakia [12]. In the 1980s, the contact and noncontact wide-angle viewing systems (WAVs) were introduced [13]. Retinal detachment repair under a microscope was suggested by Bonnet (1987). Scleral buckling was also attempted by Liu et al. (2006) [4] and Zhang et al. (2011) [11] under direct observation while utilizing a surgical microscope. Ohji and Tano (2004) [12] used a slit light and contact wide-angle lens to try scleral buckling under a microscope, and Nawrocki et al. (2008) used an optic-fiber-free intravitreal surgical system (OFFISS) [14]. In contrast to the inverted picture acquired with an indirect ophthalmoscope, this method provides an upright view of the fundus, which is advantageous. Nowadays, this technology is the most used in vitreoretinal surgery. One of the reasons why scleral buckling has become less popular among new generations of surgeons is due to a steeper learning curve with the indirect ophthalmoscopy [15]. To overcome these visual issues, a chandelier-assisted scleral buckling has been introduced, embracing the advantages that the operating microscope and modern wide-angle viewing systems offer.

The purpose of this study is to give a thorough update on chandelier-assisted scleral bucking (CSB), along with a summary of the surgical development, results, benefits, and drawbacks.

## 2. Methods of Research

**Figure 1:**
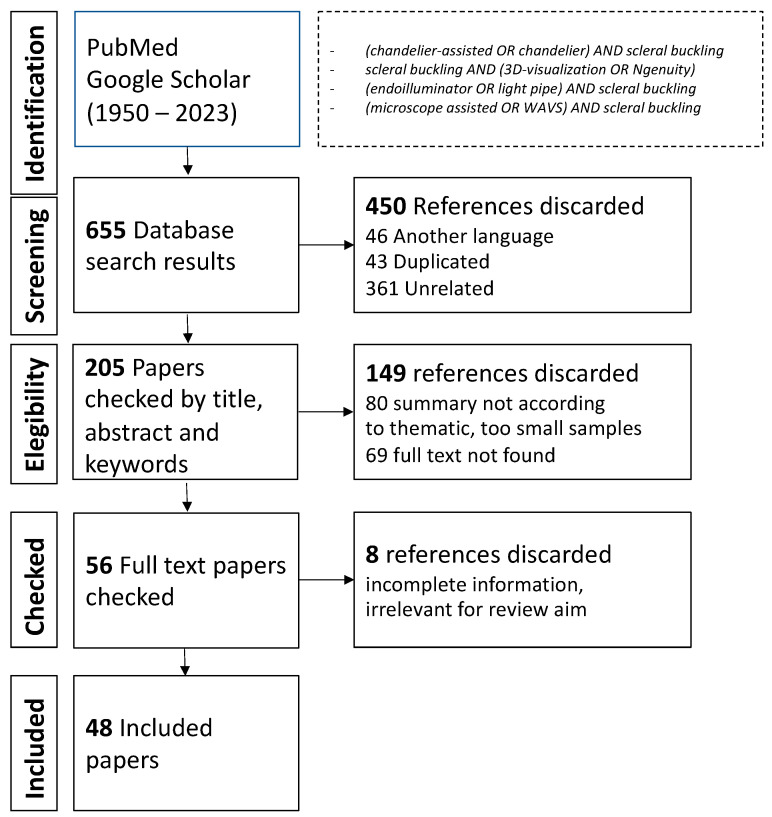
Methods of Research.

We carried out a review of the literature concerning chandelier-assisted scleral buckling using the PubMed and Google Scholar databases to February 2023 and the following MeSH terms: chandelier-assisted scleral buckling, 3D visualization, scleral buckling, scleral buckling, endoilluminator scleral buckling, microscope-assisted scleral buckling, wide-angle viewing systems (WAVs) scleral buckling, and a combination of them. We aimed at all the relevant publication in English language. We excluded studies with too-small samples of patients, except the ones that could be relevant in our opinion. No Cochrane criteria were used due to lack of randomized controlled trials on the topic. We included retrospective and prospective studies with a sample size > 10 eyes. Case reports were included only with relevant authors or surgical techniques (Figure 1).

## 3. Discussion

Nawrocki et al., in 2008, were the first to describe a sort of “endoillumination”, treating seven eyes with an operating microscope to visualize the clinical effects of scleral buckling. For fundus imaging, they used an Optic Fiber Free Intravitreal Surgical System (OFFISS) (Topcon Inc., Paramus, NJ, USA). Anatomic success was achieved in six out of seven eyes (85.7%). This method is limited so that no light fiber was put into the eye in their procedure [16].

In 2012, Aras et al. published the first description of trans-scleral fiber-optic-assisted scleral buckling for the treatment of retinal detachment (RD)using a 25-gauge torpedo-style light source (Alcon Labs, Fortworth, TX, USA). A sclerotomy without a cannula was used to deliver light from a chandelier in order to visualize the ocular fundus through a wide-angle, noncontact pars plana vitrectomy-style (PPV-style) viewing device (BIOM, OCULUS Surgical Inc., Port St. Lucie, FL, USA). Segmental or 360° circumferential buckling using a silicone sponge (506-silicone sponge; Labtician, Oakville, ON, Canada) was the technique employed for the buckling operation. The author determined the location of the chandelier based on the location of the tear. The study assessed the results of 16 eyes from 16 participants with a mean age of 53.6 ± 13.7 (19 to 73 years). Four of the eyes were pseudophakic and the others were phakic. Most of the eyes were emmetropic, with only four myopic eyes from 2.00 to 7.0 diopters. The mean follow-up time was 13.4 ± 2.8 months. The interval between the retinal detachment and surgical repair ranged from 1 day to 14 days, and the mean time was 9.6 ± 6.2 days. The preoperative visual acuity varied from 0.7 logMAR to hand movements. In 11 of the 16 eyes with subtotal retinal detachment, the macula was affected. The mean number of retinal breaks was 1.58 ± 0.7. Post-operative results showed 13 eyes (81%) had successful retinal reattachment with a single surgery. Three eyes received further pars plana vitrectomy with silicone oil injection because of failure of the previous surgery and development of proliferative vitreoretinal fibrosis (PVR) in two eyes and a sclera perforation due to excessive indentation during cryopexy in one eye. In two eyes, little choroidal bleeding occurred after choroid puncture for drainage of subretinal fluid through external sclerotomy. In four eyes, surgeons sutured the 25-gauge illumination fiber sclerotomy to avoid herniation of the vitreous. The mean best corrected visual acuity (BCVA) improved from 0.2 to 0.5 logMAR at 6 months after surgery and remained stable during the follow-up. In his study, the author claimed a better surgical view with endoillumination and a bigger vision angle (120°) compared to the OFFISS system used by Nawrocky et al. (70°) [17].

Nowadays, endoilluminator-assisted scleral buckling is more commonly performed using a 25-gauge fiber-optic chandelier light source at 3.5 and 4 mm posterior to the limbus for pseudophakic and phakic patients, respectively, through a standard trans-scleral cannula, as described by Kita et al. in 2013. In this case report, the authors showed the advantages of using a noncontact wide-angle viewing system (Resight, Carl Zeiss Meditec AG, Jena, Germany) combined with a 25-gauge light fiber illumination through a trocar, consisting of a clear image of the retina through the surgical microscope, that may be expanded and viewed even with a very small pupil, and that permits the identification of previously undetectable retinal breaks intraoperatively [18]. In 2013, Nam et al. published a work about their surgical technique for primary rhegmatogenous retinal detachment (RRD) treated with a 25 G chandelier-assisted scleral buckling and a wide-field contact lens (Mini Quad; Volk, Mentor, OH, USA). No retinal reattachment rate was shown. Authors highlighted the advantages of chandelier light compared to the torpedo light of Aras et al., such as the angle of scleral incision using the trocar blade that leads to less vitreous leakage and thus less need of scleral suturing. The author placed the chandelier 90° away from the tear position. Further, authors stressed the greater safety of the light probe inside the eye compared to the torpedo-style, as chandelier-related complications were not present [19].

In the same year, Nagpal et al. prospectively studied 10 patients who underwent SB using a wide-field contact lens viewing system with a single 25-gauge chandelier for illumination. Out of 10 patients, there were 7 phakic and 2 pseudophakic eyes, and 1 aphakic eye. The retinal detachment (RD) affected more than two quadrants in five of the eyes (50%).Total retinal detachment (RD)was found in four eyes (40%). In one eye (10%), the macula had been spared in the retinal detachment (RD). Eight eyes were encircled with a scleral band, and two eyes had segmental buckles. In all cases, subretinal fluid was evacuated. At 6 months follow-up, 9 of the 10 eyes had a complete retinal reattachment. The preoperative mean best corrected visual acuity (BCVA) of 1.3 logMAR improved with surgery to 0.48 at 6 months. The author claimed that the procedure takes the same amount of time as the typical indirect ophthalmoscopy-based surgery. During surgery, the author preferred to remove the light probe and plug the cannula while manipulating the eye and reinserting only when needed.

Interestingly, he compared his work with the other previous studies, justifying the choice of a contact lens (Volk HRX Vit SSV) to obtain a wider view (150°) compared to Nawrocky et al. (50°) and minimal distortion in the periphery during indentation because of the lens adhesion on the cornea, which is present in noncontact-based systems such as in the Aras et al. study. The author did not mention any chandelier-related complications, although he stressed the higher risk of endophthalmitis [20].

In 2014, Gogia et al. evaluated the surgical outcomes of endoillumination-assisted scleral buckling (EASB) with the use of a 25-gauge “self-retaining endoilluminator”, through the Volk Reinverting Operating Lens System and wide-angle viewing system wide-angle viewing systems (WAVs) to treat a group of 25 phakic and pseudophakic patients with retinal detachment (RD)and proliferative vitreoretinopathy (PVR) grade < C2, whose retinal breaks could not be detected before surgery. The median age was 46 years (range: 17–72 years). In two patients, a pars plana vitrectomy was required because breaks were not detected even using the chandelier. Of the 23 eyes that underwent EASB successfully, 22 (95.6%) had complete retinal attachment following a single surgery, and at the end of follow-up, mean visual acuity was 1.09 ± 0.46 logMAR compared with 1.77 ± 0.28 logMAR preoperatively. No patient had any complications during the two-year follow-up [21].

In children, retinal detachment (RD) accounts for only 1.7–12.6% of all cases. It is distinguished by a high incidence of macular involvement and proliferative vitreoretinopathy (PVR) at presentation. Except in the presence of severe PVR, opaque media, or an undetectable break, the scleral buckling procedure is preferred in pediatric retinal detachment (RD) patients. In 2015, twin un-cannulated 27-gauge chandeliers (Eckard TwinLight Chandelier; DORC International, Zuidland, The Netherlands) and a standard vitrectomy contact lens (HHV Dispo; Hoya, Tokyo, Japan), or a noncontact wide-angle viewing system were adopted by Yokoyama et al. in three pediatric patients (7 years, 12 years, and 11 years). All patients had retinal detachment (RD) with macular involvement, and small retinal holes, of which two were preoperatively undetectable. The authors, after identifying the retinal breaks, decided to remove the light source in order to perform subretinal fluid drainage or scleral devices suture. In two cases, retinal tears not observed preoperatively were discovered intraoperatively. All three patients had improved visual acuity, permanent retinal reattachment, and no problems. At the end of surgery, the 27-gauge sclerotomies were completely covered by conjunctiva and were not sutured. Retinal reattachment was achieved in all cases without intra- or postoperative complications. The authors stressed the difficulty of fundus examination using the ophthalmoscope in pediatric patients, thus making scleral buckling with endoillumination a useful tool to localize and treat retinal breaks [22].

In 2015, a retrospective comparation between patients who underwent chandelier-assisted scleral buckling (CSB) with noncontact wide-angle viewing and a group that underwent standard scleral buckling (SSB) with the indirect ophthalmoscope was published by Narayan et al. The mean age of patients was 32.48 ± 18.41 and 32.50 ± 17.11 years in the CSB and SSB group, respectively. Both groups showed a similar rate of retinal reattachment: 13 of 14 eyes (92.85%) in the chandelier-assisted scleral buckling (CSB) group and 12 of 14 eyes (85.71%) in the standard scleral buckling (SSB) group, with a shorter mean surgical time in the first group (77.85 ± 16.37 min) compared with the second (95.71 ± 26.59 min, *p* = 0.037). One patient in each group showed an increase in intraocular pressure. In the SSB group, one patient developed proliferative vitreoretinopathy (PVR), while three patients in the chandelier-assisted scleral buckling (CSB)group experienced intraoperative leakage at the chandelier insertion site. In all these cases, sclerotomies were carefully sutured with vicryl 7/0 before conjunctival closure. One eye in the chandelier-assisted scleral buckling (CSB)group and two eyes in the standard scleral buckling (SSB) group required vitrectomy for recurrent retinal detachment (RD). The mean visual acuity (Snellen visual acuity assessment) improved from 20/160 to 20/80 in the CSB group, and 20/320 to 20/160 in the standard scleral buckling (SSB) group. The authors highlighted the increased cost of chandelier-assisted scleral buckling (CSB) compared to standard scleral buckling (SSB), but the apparent reduced surgical time saves indirect cost [23].

In the same year, Imai et al. published a retrospective study in which 79 patients with a mean age of 43.7 ± 16.0 years, treated by two different surgeons, with first-ever retinal detachment underwent scleral buckling using a 25 G cannulated chandelier and noncontact wide-field viewing system. The success rates were retrospectively 92.4% and 100%. Final best corrected visual acuity (BCVA) was 0.10 ± 0.31 logMAR units, significantly better than preoperative best corrected visual acuity (BCVA) (0.31 ± 0.65 logMAR units) (*p* = 0.0007, paired *t*-test). The mean number of retinal breaks was 1.5 ± 0.8. The position of the trocar cannula was determined by the location of the break. retinal detachment (RD)affected one or two quadrants in twenty-eight eyes. Fifty-one eyes exhibited more extensive retinal detachment (RD), with involvement in three or four quadrants. In 43 (54.4%) of the eyes, the macula was uninvolved prior to surgery. Fifty-three eyes had external subretinal fluid drainage, whereas the remaining twenty-six did not. Twenty-one individuals were subjected to segmental buckling, whereas fifty-eight had 360° circumferential buckling. Despite the results, five choroidal hemorrhages, one iatrogenic retinal break, and one lens touch by the chandelier tip occurred during surgery. Six cases of retinal re-detachments were reported during the follow-up period of approximately 11.8 ± 6.9 months. One eye developed secondary glaucoma and one eye a cataract progression [24].

Seider et al. achieved a primary reattachment in 10 of 12 patients without chandelier related complications. A single valveless pars plana 25-gauge cannula/chandelier system was utilized in eleven eyes, and a dual 29-gauge chandelier/cannula system (Synergetics, O’Fallon, MI, USA) was used in one eye. The noncontact BIOM or contact Volk system (Volk Optical Inc., Mentor, OH, USA) were used to enable wide-angle view via the microscope. In the two failures, a reattachment was achieved after a single PPV. No additional retinal breaks were discovered around the prior chandelier implantation in either patient. Any vitreous prolapse that was visible after the cannula was removed was cut with scissors. The sclerotomy was sutured soon after the cannula was removed in all cases. The author stated that the use of the chandelier did not seem to prolong the duration of surgery (mean 117.9 min, range 46–149 min) [25,26].

Tomita et al. in 2015 reviewed retrospectively the outcomes of 16 patients (mean age 41.3  ±  14.0 years; range 24–65 years) treated with scleral buckling using a wide-angle viewing systems (WAVs) and 23 patients (mean age 45.3  ±  12.5 years; range 23–68 years) treated with conventional indirect ophthalmoscopy. A 25-gauge endoilluminator was used. The mean postoperative follow-up was 10.4 months (range 6–24 months). There were no significant differences between the two regarding demographics data. Only one eye was pseudophakic in the wide-angle viewing systems (WAVs) group. No significant differences were observed regarding retinal reattachment (wide-angle viewing systems (WAVs) 93.8%, conventional 95.7%, *p*  =  0.79), postoperative best corrected visual acuity (BCVA) (*p*  =  0.28) and complications rate. On average, no significant difference in the overall surgical time between the wide-angle viewing systems (WAVs) and conventional groups (*p*  =  0.07) but was shorter using a noncontact wide-angle viewing systems (WAVs) (*p*-value 0.02) in cases that underwent segmental buckling (92 ± 33 min in the wide-angle viewing systems (WAVs) group compared to the 117  ± 34 min in the conventional group). Interestingly, five patients needed corneal epithelial peeling due to corneal edema in conventional surgery, while no patients had this complication in wide-angle viewing systems (WAVs). This was possibly because the wide surgical view provided by the noncontact wide-angle viewing systems (WAVs) allowed for easier observation of the retinal tears with less scleral indentation, resulting in a lesser rise in intraocular pressure and less strain on the cornea. The viscoelastic material placed on the cornea during noncontact wide-angle viewing systems (WAVs) may also have contributed to the reduction in anterior surface damage. No viscoelastic was used in the conventional group. Other non-significant postoperative complications were macular oedema and subfoveal serous detachment in two eyes treated with standard scleral buckling, while macular pucker and faster cataract progression in two eyes treated with noncontact wide-angle viewing systems (WAVs) and endoilluminator [27].

In 2016, Haug et al. presented a new surgical technique in seven patients (mean age was 52 years range: 28–65 years old) with retinal detachment (RD) (in two cases, the macula was involved). All patients were operated under conventional indirect ophthalmoscopy for identification and cryotherapy, but a 25-gauge chandelier trocar-based cannula with assisted noncontact wide-angle view was used during subretinal fluid drainage. In six out of seven patients, retinal reattachment was achieved. No complications were reported. The mean follow-up time was 6 months. No functional results were provided [28].

Hu et al. in 2017 published a retrospective study of 61 eyes (aged 12–67 years) with retinal detachment (RD) treated with scleral buckling using 25-gauge chandelier endoillumination under surgical microscope. A corneal ring was used for setting a 30° or 50° Landers contact prism lens. This kind of contact lens does not require an image converter on the microscope. Follow-up time ranged from 6 to 27 months. Circular buckle surgery was performed on 21 eyes, segmental buckle on 40 eyes, subretinal fluid drainage on 22 eyes, and air injection on 5 eyes. The author placed the chandelier in the inferior quadrants opposite to the nasal or temporal area of breaks localization. Of all patients, 93.4% had retinal reattachment with one surgery. Four cases of retinal detachment recurrence were observed, of which three were due to PVR and one to new retinal tears. All patients were monitored for intraocular hypotony on day 1, 3 and 7. No hypotony was observed. Scleral incision was sutured in all patients to prevent vitreous leakage. A post-operative ultrasound biomicroscope (UBM) examination showed no vitreous incarceration at 1 and 3-months. The only complication observed was a mild subretinal hemorrhage which occurred in 2 eyes during subretinal fluid drainage. No lens damage, vitreous hemorrhage or retinal injury were observed due to chandelier insertion [14].

In 2017, Jo et al. reported the outcomes of 17 phakic eyes in 16 patients (mean age of 26.8 ± 10.2, range, 11 to 47 years) using a wide-angle viewing systems (WAVs) scleral buckling with a 25-gauge chandelier endoilluminator. In this article the author made a nice comparison with previous studies cited in this review, stressing the advantages concerning operative time. Mean preoperative best corrected visual acuity (BCVA) was 0.23 ± 0.28 logMAR, while post-operative at the end of follow-up was 0.20 ± 0.25 (*p* = 0.722). The mean postoperative follow-up period was 7.3 ± 3.1 (range, 3 to 12) months. The mean operative time for all patients was 76.8 ± 16.1 min, with an anatomical success rate of 94.1% (16/17 patients). The re-detachment was treated with PPV. In seven eyes, retinal breaks were found in the superior retina, eight in the inferior retina, and two in the temporal retina. The chandelier’s placement was defined by the position of the retinal break, which might be 90° or 180° away from the break. Five eyes had retinal detachment (RD)in one quadrant, nine eyes had retinal detachment (RD)in two quadrants, and three eyes had more severe retinal detachment (RD)in three or four quadrants. Six patients had segmental buckling and 11 patients had encircling buckling. One eye had loss of vitreous through the scleral port, while 4 eyes had post-operative increased intraocular pressure (IOP) ≥ 25 mmHg and one eye had herpes simplex epithelial keratitis [29].

In the same year, Assi et al. published a prospective observational study using a 23-gauge endoillumination probe and a noncontact wide-angle viewing operating system in 23 eyes with a 1 year follow-up. Mean age was 26.7 ± 11.5 years. Only 1 eye was pseudophakic. The retinal detachment (RD)compromised one quadrant or fewer in two (9%) eyes, one to three quadrants in seventeen (74%), and more than three quadrants in four (17%) eyes. The macula remained flat in 7 (30%) of the eyes and detached in the others. A retinal dialysis was discovered in 9 (39%) patients, one retinal hole in 5 (22%) patients, two retinal holes in 4 (17%) patients, and three or more retinal holes in the remaining patients. The self-sealing cannula of the probe was placed opposite the retinal breaks. Intraocular gas or air was injected as needed during the procedure to restore IOP. Retinal reattachment was achieved in 20 patients (87%), while 1 patient needed 2 additional surgeries and 2 patients required 3 more surgeries. All patients had a flat retina at 1 year. Silicon oil was present in one eye. Mean best corrected visual acuity (BCVA) improved from 1.03 ± 0.83 logMAR preoperatively to 0.40 ± 0.47 logarithm at 1 year. Six patients (26%) developed posterior vitreous detachment. Six eyes (26%) required suture of the sclerotomy with a vicryl 8.0. No further complications related to chandelier use were observed [30].

To reduce the risk of vitreous traction during trocar insertion as well as lens damage and endophthalmitis, Caporossi et al. used in a retrospective case review a 27-gauge intraocular endoilluminator fiber through a valved trocar cannula and a noncontact wide-angle viewing systems (WAVs) (Resight 700; Carl Zeiss Meditec AG) in 28 eyes of 28 patients (mean age 61.5 years; range 37–78 years). Four eyes were pseudophakic and 24 were phakic. The position of the trocar cannula was chosen depending on the localization of the break. There was one retinal break in 21 eyes, two in 5 cases, 3 in one eye, and 4 in another one (mean 1.35 ± 0.73). During surgery, hidden retinal breaks were discovered in two eyes of two pseudophakic patients. The mean extent of the retinal detachment was 5.3 ± 2.5 clock hours (range 1–12 h). Two eyes exhibited total retinal detachment, with one retinal break in the superior quadrants. Both patients were phakic. Ten eyes had a retinal detachment that extended more than 6′ clock hours. In 18 of the eyes (64.28%), a radial buckle was employed. A circumferential buckle was implanted in nine eyes (32.14%), while one eye (3.57%) received both a radial buckle and a circumferential buckle for multiple retinal breaks. There was no encircling band employed. To achieve adequate scleral indentation, 18 eyes (64.28%) underwent evacuation drainage with intravitreal air injection because of an increase in subretinal fluid. The patients were followed up for an average time of 6.4 months and all patients showed statistically significant improvement in best corrected visual acuity (BCVA): mean postoperative best corrected visual acuity (BCVA) was 0.22 ± 0.25 logMAR compared to mean preoperative best corrected visual acuity (BCVA) of 0.8 ± 0.88 logMAR. Only one patient required an additional vitrectomy with silicone oil tamponade due to development of proliferative vitreoretinopathy. No suture at the sclerotomy site was needed and no complications related to the use of the endoilluminator have been reported. The author stresses many advantages of the 27-gauge cannula, including a more focused light and the lower risk of complications. An interesting possibility described by the author is to inject air directly through the trocar into the vitreous cavity during the subretinal fluid drainage that results in a homogeneous bubble of air and avoids the fish-egg effect [31].

In 2021 Nam et al. described a successful case report of scleral buckling using a 27-gauge light pipe through valved cannula and a wide-field contact lens (Mini Quad; Volk, Mentor, OH, USA) which proved to be easier in terms of light direction adjustment compared to a chandelier-assisted surgery [32].

In 2019, Cohen et al. made a retrospective case series of 49 eyes. Out of these 49 eyes, 27 were treated with traditional scleral buckling (TSB) (mean age of 47.56 ± 16.47), while 22 with chandelier-assisted scleral buckling (CSB) (mean age 35.73 ± 15.34) with no significant difference in terms of reattachment rate 85.2% (23 of 27 eyes) in the TSB group and 81.8% (18 out of 22 eyes) in the CSB group, (*p* = 1.00). In CSB, they used a wide-angle viewing system (BIOM, Port St. Lucie, FL, USA) along with a chandelier 25-gauge light source. During the placement of scleral sutures, buckle insertion, and subretinal fluid drainage, the 25-gauge cannula was closed with a scleral plug. In both groups, there were 16 macula-on retinal detachments and multiple retinal tears (12 in CSB and 8 in TSB). Scleral buckling was the first surgery for 19 patients in the CSB group and 21 patients in the TSB group. In three eyes of each group, PPV was required for retinal reattachment. At 6 months, mean best corrected visual acuity (BCVA) was 0.245 ± 0.30 in the CSB group and 0.368 ± 0.34 in the traditional scleral buckling (TSB)group, but the difference was not statistically significant (*p*= 0.229). An interesting datapoint is the rate of retinal tear identification in traditional scleral buckling (TSB)and chandelier-assisted scleral buckling (CSB), where they found additional intraoperative retinal tears in 7 out of 22 (31.8%) of the eyes in the chandelier-assisted scleral buckling (CSB) group, compared to 5 out of 27 (18.5%) in the traditional scleral buckling (TSB)group. Overall, there was a 71.9% difference in the rate of intraoperative retinal-break discovery. One case in the chandelier-assisted scleral buckling (CSB) group had a subretinal hemorrhage, while two eyes in each group developed cataracts, but none of them required surgery during the follow-up period of 6 months [33].

In 2019, Nossair et al. conducted a retrospective non-comparative study of 21 consecutive eyes with uncomplicated pseudophakic retinal detachment (RD) treated with chandelier-assisted scleral buckling (CSB)using a 25-gauge chandelier endoilluminator inserted 180° away from retinal breaks with a wide-angle viewing systems (WAVs) contact lens (Mini Quad; Volk, Mentor, OH, USA). In five eyes (23.81%), a segmental tire alone was used, while in seven eyes (33.33%), this was combined with an encircling band. In three eyes (14.29%), a radial sponge alone was used, while in six eyes (28.57%), this was combined with an encircling band. The chandelier scleral wound was sutured using 8.0 vicryl. The mean follow-up period was 24.09 ± 10.78 months (range 6–42 months). Primary retinal reattachment was gained in 19 eyes (90.48%), while PVR caused retinal re-detachment in two (9.52%) eyes. Intraoperative complications included vitreous leakage at the chandelier sclerotomy site in four eyes (19.05%), and localized subretinal hemorrhage at the drainage site in one eye (4.76%). Choroidal detachment in one eye (4.76%) and increased intraocular pressure in two eyes (9.52%) were early postoperative complications; epiretinal membrane development in one eye (4.76%), extraocular muscle disorder in one eye (4.76%), and proliferative vitreoretinopathy in three eyes (14.29%) were among the late postoperative problems. The mean corrected distance visual acuity following surgery was 0.7 ± 0.3 logMAR units [34].

In 2016, the introduction of the newest 3D heads-up visualization system provided a new tool in ophthalmic surgery, especially for vitreoretinal surgery. Improved ergonomics, easier surgical education and better manipulation due to an enhanced visualization are the most important benefits. The advantages of the 3D chandelier-assisted scleral buckling surgery were assessed in 2019 by Kita et al. in a case series of 18 eyes. The NGENUITY 3D visualization system (Alcon Laboratories, Fort Worth, TX, USA), which was connected to a VISU 210 microscope (Carl Zeiss Meditec, Jena, Germany), was used to watch all surgical operations. The intraocular lighting system was a 27 G mono chandelier light system by DORC. Retinal reattachment was achieved in all cases, with a mean follow-up time of 11.3 months. Except for postoperative diplopia in one case that underwent supplemental radial buckling for a deep retinal tear, there were no intraoperative or postoperative complications in the patients. Interestingly, with the aim of providing a good-quality intraoperative view without the second operator having to carry out a continuous hydration, they used a particular type of contact lens previously developed by the same authors (HOYA Corporation, Tokyo, Japan) [35].

In 2020, Frisina et al. carried out a retrospective study of 213 eyes of 205 patients to report the outcomes of ab externo surgery using a surgical microscope and wide-angle viewing system (WAVS). Different chandelier endoilluminators with a gauge of 25, 27, or 29 were used. The surgical time was 75.5 ± 42 min (range 30–115). This surgery time confirms exactly the findings published in the literature. During the follow-up, established at 3 months, six eyes (2.8%) developed a recurrent retinal detachment (RD), three of which were caused by proliferative vitreoretinopathy and three of which were caused by a new retinal tear. After one week, one eye developed a macular hole, while two eyes experienced a vitreous hemorrhage. Pars plana vitrectomy (PPV) was used to treat every patient. No iatrogenic damage of the lens was observed. Vitreous leakage after removal of the chandelier through sclerotomy was observed in five cases with a 25-gauge, four cases with a 27-gauge, and three cases with a 29-gauge. According to them, the size of the sclerotomy has no bearing on the vitreous leakage. It may be caused by two things: first, the sclerotomy construction technique, which emphasizes the need to insert the trocar obliquely rather than perpendicular to the sclera; and second, the abnormalities of the vitreous (synchisis, syneresis), which can influence vitreous leakage independently of the size of the sclerotomy [36].

Yannuzzi et al., in 2020, reported a modified chandelier-assisted scleral buckling technique in three patients with retinal detachment using an illuminated endolaser probe, in addition to or as a substitute for cryotherapy for retinopexy. As a matter of fact, the authors demonstrated how the use of the endolaser could easily overcome the difficulty and the limitations of performing cryopexy in the case of a posterior break or when its localization is directly beneath the recti muscles. The laser probe is inserted through the same trocar of the chandelier illuminator. No iatrogenic retinal breaks were provoked by endolaser insertion. At 6 months, the retina remained attached in all patients and no complications were observed. The use of the endolaser in endoillumination-aided scleral buckling for the treatment of rhegmatogenous retinal detachment is found to be both safe and successful in this small case series [37].

One limit of chandelier endoillumination is the poor ergonomics and mobility that it offers, which is why Agranat et al., in 2020, developed a new technique using a 25 or 27-gauge guarded light pipe and the Ngenuity digital three-dimensional platform. The vitrectomy light probe is modified with a trimmed Watzke–Allen sleeve, in order to expose only 5 mm of the light pipe and avoid dangerous vitreal insertion. This allows a thorough fundus exploration with better ergonomics. Although they only have limited experience with the technique they described, so far there have been no unfavorable outcomes or events [38].

In 2022, Baldwin et al. compared 47 eyes that underwent scleral buckling (SB) for the repair of rhegmatogenous retinal detachment (RRD) with either traditional indirect ophthalmoscopy (ID) (*n* = 31) or guarded light pipe with the Ngenuity 3D vision system (3DGLP) (*n* = 16) in a retrospective comparison analysis. They evaluated anatomic success, post-operative visual acuity, operative time, and complications. The study showed an 87.5% anatomic success rate with a single surgery in 3D with a guarded light pipe (3DGLP) compared to 87.0% with traditional scleral buckling. Median post-operative visual acuity was 0.10 (0.0–0.20) in the traditional scleral buckling group and 0.08 (0.02–0.69) in the 3D with a guarded light pipe (3DGLP) group (*p* = 0.51). The median surgery time was 107 (94–123) minutes in the traditional scleral buckling group and 100 (90–111) minutes in the 3D with a guarded light pipe (3DGLP) group (*p* = 0.25). The traditional scleral buckling group had no post-operative problems, but the 3D with a guarded light pipe (3DGLP) group experienced one self-resolving vitreous hemorrhage issue linked to a broken cryoprobe (*p* = 0.34). In neither group were there any instances of post-operative cataract advancement [39].

In 2020, Savastano et al. described a new variant of chandelier-assisted scleral buckling (CSB) using a 27-gauge chandelier and 7.0 vicryl releasable scleral sutures in 20 eyes of 20 patients. A primary success rate was reported in 19 out of 20 eyes at 6 months. None of the surgically repaired eyes had lens opacity or scarring at the location of the chandelier’s entry, and no cataract development was seen during the follow-up. There were no reported difficulties during the procedure or postoperative complications. According to a prior publication, the main reattachment results of the EI chandelier-assisted SB approach and traditional indirect ophthalmoscopic SB surgery are comparable. With this technique, the authors claim to reduce the risks linked to the creation of a sclerotomy, and thereby it may play a role in reducing the unwillingness of some ophthalmologists to perform scleral buckling (SB) surgery. The authors do not recommend using a valved cannula for the same purpose, because the chandelier tip is smaller and, in some designs, may be shortened to the bare minimum length required, whereas the cannula has a fixed-length intraocular metallic part that may potentially touch the lens while manipulating the eye. Moreover, the cannula would need to be withdrawn and a suture would need to be applied at the time the buckle was inserted. This can potentially widen the sclerotomy site and raise the possibility of postoperative problems [40].

A modified procedure utilizing a 25-gauge valved trocar in 107 eyes was proposed by La Spina et al. in 2021 after a retrospective review. The valved trocar permits the removal of the chandelier with a reduced risk of vitreous prolapse. The chandelier can now be removed and reinserted as necessary during the procedure. This lowers the risk of accidental lens traumas, iatrogenic hypotony, choroidal hemorrhage, or choroidal detachment. The retinal reattachment rate was 94% (101 eyes) at 3 months. In their paper, they also emphasize the importance of this technique, accompanied by the use of a digital three-dimensional (3D) visualization system, the Ngenuity 3D vision system coupled with an OPMI Lumera 700 surgical microscope, and a Resight wide-angle viewing systems (WAVs), in terms of reducing the surgical complexity of a traditional SB and by doing so, making it more accessible even to less experienced surgeons [41].

In 2022, Albalkini et al. compared the anatomical and functional outcomes and rate of complications of standard scleral buckling (SSB) versus chandelier-assisted scleral buckling (CSB) in 50 phakic eyes with rhegmatogenous retinal detachment. They stated that chandelier-assisted scleral buckling (CSB) did not achieve consistent advantages over SSB, but rather could lead to a greater rate of postoperative complications. The outcomes were not statistically different, while the surgical time was in favor of chandelier-assisted scleral buckling (CSB) (120.3 ± 39.05 and 102.48 ± 43.76 min for the standard scleral buckling (SSB and chandelier-assisted scleral buckling (CSB), respectively, *p* = 0.1). Interestingly, the rate of complications were higher in chandelier-assisted scleral buckling (CSB) (34.8% vs. 3.8%, *p* < 0.05), with a more frequent rate of epiretinal membranes (44% vs. 19%, *p*< 0.05) and vitreous incarceration in the chandelier sclerotomy site [42].

## 4. Conclusions

The use of chandelier-assisted scleral buckling (CSB) appears to be supported nowadays in the literature due to its versatility and easier learning curve. Despite the use of chandelier does not change the principles of scleral buckling surgery, many authors in the current literature have received benefits to develop surgical variants to make the surgery easier and safer (Table 1). One of the prognostic indicators for poor surgical results in rhegmatogenous retinal detachments is the failure to identify retinal breaks. The results in terms of retinal reattachment seems to be comparable to standard scleral buckling but with a higher risk of iatrogenic damage. Indirect ophthalmoscopy remains a challenging surgical visual system that gives an inverted image and handling needs experience. Further, some cases may be more difficult than other due to patients with small pupils or lens opacification. The main advantages of using an operative microscope with chandelier illumination are consequently better surgical visualization that results in an easier and a more standardized surgery, with improved ergonomics compared to indirect ophthalmoscopy. In several studies, the authors reported that they could find previously unidentified retinal breaks intraoperatively [18,22]. Further, the majority of authors agree that chandelier-assisted scleral buckling (CSB) is a powerful training tool for young surgeons and an excellent aid in clinical studies.

A recent study showed that when performing SB, 3D surgery proved safe and offered non-inferior results to conventional microscope viewing [43]. Surgical time reduction seems another valuable feature in chandelier-assisted scleral buckling (CSB) compared to conventional SB [44]. Although chandelier insertion changed SB from an extraocular to an intraocular procedure, there is rarely evidence of complications related to this procedure, such as IOL displacement, iatrogenic break [24], retinal incarceration, vitreous prolapse, or endophthalmitis [45]. Regarding vitreous prolapse, the majority of authors decided to suture the sclerotomy with vicryl 7/0 or 8/0 at the chandelier insertion site, even if no vitreous wick was observed. Cataract progression is a possible complication, as shown in three studies in this review. Cataract development appears to be a downside of chandelier-assisted scleral buckling (CSB), since the use of scleral buckling (SB) is one of the main indications in young patients in order reserve the lens. However, because of the lack of large prospective studies, it is difficult to state an evidence-based superiority in terms of results, surgical time, and complications compared to conventional scleral buckling. The currently available comparative studies present several limitations, such as the heterogeneity of methods and samples.

## 5. Future Directions

With this review, we would like to stress that scleral buckling is wrongly considered a declining procedure in favor of pars plana vitrectomy (PPV). Scleral buckling (SB) has the benefit of being an extraocular operation that is more forgiving in the event of failure than vitrectomy, since proliferative vitreo-retinopathy (PVR) develops more quickly in situations where vitrectomy was the primary procedure. Chandelier-assisted scleral buckling (CSB) seems to be more an evolution of standard scleral buckling (SB) toward a more feasible surgery for all surgeons. Despite modifying standard scleral buckling (SB) from extraocular to an intraocular procedure, it minimize the risk of iatrogenic damage through less invasive ways to use the chandelier, such as smaller port and improved optical fibers. The 3D-visualization systems are rising in popularity and we think that in the near future the use of artificial intelligence could be another tool to aid the next generations of surgeons in scleral buckling surgery [46].

## Figures and Tables

**Table 1:** Main preoperative results and outcome of chandelier-assisted scleral-buckling-related articles.

Author, Year	Study Design	Eyes, N.	Preop BCVA (Mean ± SD) logMAR ^†^	Postop BCVA (Mean ± SD) logMAR ^†^	Technique	Operative Time (min)	Reattachment Rate N, (%)	Complications N. Eyes (%)	Final Follow-up, Months (M) or Days (D)
Aras et al. (2012) [17]	Prospective study	16	0.2 ± NA	0.5 ± NA	25 G trans-scleral fiber-optic-assisted SB with a torpedo-style light source noncontact WAVS	NA	13 (81.3)	- GP, 1 (6.25) - rRD, 2 (12.5) - Vitreous prolapse at the chandelier insertion site, 4 (25)	13.4 M
Kita et al. (2013) [18]	Case Report	1	0.7	0.9	25 G CSB with noncontact WAVS	NA	1 (100)	None	6 M
Nam et al. (2013) [19]	Surgical Technique/Case Series	12	NA	NA	25 G CSB and a WAVS contact lens	NA	NA	None	NA
Nagpal et al. (2013) [20]	Prospective study	10	1.3 ± NA	0.48 ± NA	25 G CSB with contact WAVS	NA	9 (90)	rRD, 1 (10)	6 M
Gogia et al. (2014) [21]	Prospective study	25	1.77 ± 0.28	1.09 ± 0.46	25 G CSB with contact WAVS	NA	22/23 (95.6)	PPV performed where break was not localized intraoperatively, 2 (8) (no suture?)	24 M
Yokoyama et al. (2017) [22]	Case Series	3	for single case Case 1: 0.3 Case 2: 0.2 Case 3: 0.2	for single case Case 1: 0.4 Case 2: 0.8 Case 3: 0.6	27 G twin CSB with contact/noncontact WAVS	NA	3(100)	None	NA
Narayanan et al. (2016) [23] **	Retrospective study	CSB group: 14 **	0.9 ± NA	0.6 ± NA	25 G CSB with noncontact WAVS and Standard SB	77.85 ± 16.37	13 (92.8)	- Increase in IOP, 1 (7.1) - Vitreous prolapse at the chandelier insertion site, 3 (21,4) - PPV for rRD, 1 (7.1)	186.85 ± 115.63 D(range: 1–11 M)
Imai et al. (2015) [24]	Retrospective study	79	0.31 ± 0.65	0.10 ± 0.31	25 G CSB with noncontact WAVS	100.3 ± 31.3	96.2 *	- CH, 5 (6.3) - New retinal break, 1 (1.3) - Lens touch by the chandelier, 1 (1.3) - rRD, 6 (7.6) - Secondary glaucoma, 1 (1.3) - Cataract, 1 (1.3)	11.8 ± 6.9 M
Seider et al. (2015) [25,26]	Retrospective study	12	0.2 ± NA	0.1 ± NA	25 G CSB (11 eyes) and 29 G CSB (1 eye) with contact or noncontact WAVS	117.9 (46–149)	12 (100)	- Cataract, 2 (16.7)	234.6 (90–455) D
Tomita et al. (2015) [27] **	Retrospective study	WAVS group: 16 **	0.01 ± 0.24	0.04 ± 0.08	25 G CSB with noncontact WAVS and standard SB	107 ± 41	WAVS group: 15 (93.8)	- ERM, 1 (6.3) - Corneal epithelial disorder, 1 (6.3)	10.4 M
Haug et al. (2016) [28]	Surgical Technique/Case series	7	NA	NA	25 G CSB with noncontact WAVS	NA	6 (85.7)	- PPV for rRD, 1 (14.3)	6 M
Hu et al. (2017) [14]	Prospective study	61	NA	NA	25 G CSB with contact WAVS	NA	57 (93.4)	- SH: 2 (3.3) - rRD, 4 (6.6)	6–27 M
Jo et al. (2017) [29]	Retrospective study	17	0.23 ± 0.28	0.20 ± 0.25	25 G CSB with noncontact WAVS	76.8 ± 16.1	16 (94.1)	- Vitreous prolapse at the chandelier insertion site, 1 (5.9) - Increased IOP, 4 (23.5) - Herpes simplex epithelial keratitis, 1 (5.9) - PPV for rRD, 1 (5.9)	7.3 ± 3.1 M range (3–12 M)
Assi et al. (2018) [30]	Prospective study	23	1.03 ± 0.83	0.40 ± 0.47	23 G endoilluminator probe with noncontact WAVS	NA	20 (87)	- PVR, 2 (8.7) - Extension of the buckle for new breaks, 1 (4.3)	12 M
Caporossi et al. (2019) [31]	Surgical Technique/Case Series	28	0.8 ± 0.88	0.22 ± 0.25	27 G CSB with noncontact WAVS	NA	27 (96.4)	- PVR, 1 (3.6)	6.4 M
Nam et al. (2021) [32]	Case Report	1	NA	NA	27 G endoilluminator probe with contact WAVS	NA	1 (100)	None	1 M
Cohen et al. (2019) [33] **	Retrospective study	22	0.5 ± NA	0.25 ± 0.30	25 G CSB with noncontact WAVS and Standard SB	NA	18 (81.8)	- Cataract, 2, (12.5)	6 M
Nossair et al. (2019) [34]	Retrospective study	21	1.28 ± 0.41	0.7 ± 0.30	25 G CSB with contact WAVS	NA	19 (90.48)	- Vitreous prolapse at chandelier insertion site, 4 (19.05) - Localized SH at drainage site 1 (4.76) - Choroidal detachment, 1 (4.76) - Elevated IOP, 2 (9.52) - ERM, 1 (4.76) - PVR, 3 (14.29) - rRD, 2 (9.52%)	24 M
Kita et al. (2019) [35]	Retrospective study	18	0.28 ± 0.36	0.07 ± 0.11	3D, 27 G chandelier SB with contact/noncontact WAVS	NA	18 (100)	- Diplopia, 1 (5.55)	11.3 M
Frisina et al. (2019) [36]	Retrospective study	213	0.46 ± 0.76	0.2 ± 0.2	25 G 27 G 29 G CSB with noncontact WAVS	75.5 ± 42	207 (97.19)	- rRD 6 (2.81) secondary to PVR (3 eyes) and to a new retinal break (3 eyes) - Persistent VH2 (0.93) - MH 1 (0.46)	3 M
Yannuzzi et al. (2020) [37]	Case series	3	NA	NA	25 G CSB with noncontact WAVS and endolaser illuminated probe	NA	3 (100)	None	6 M
Agranat et al. (2020) [38]	Surgical Technique	NA	NA	NA	3D, 25 G or 27 G modified endoilluminator probe with a silicone guard, with noncontact WAVS	NA	NA	NA	NA
Baldwin et al. (2019) [39] **	Retrospective study	16	0.03 ± NA (range 0–0.18)	0.08 ± NA (range 0.02–0.69)	3D, 25 G or 27 G Guarded Light Pipe Endoillumination noncontact WAVS and standard SB	- 3D: 90–111; 86–111 (with SRF drainage) - Standard SB: 94–123; 100–135 (with SRF drainage)	14 (87.5)	VH 1 (6.3%)	median 11.6 (5.0–23.2)M
Savastano et al. (2020) [40]	Retrospective study	20	0.23 ± 0.29	0.77 ± 0.17	27 G CSB with noncontact WAVS and releasable 7.0 vicryl scleral suture	NA	19(95)	- rRD 1	6 M
La Spina et al. (2021) [41]	Retrospective study	107	NA	NA	3D, 25 G CSB viewing system with noncontact	Mean 41 ± 18	(4)	- Displacement of a radial scleral implant 1 (0.9%) -rRD 5 (6%)	3 M
Albalkini et al. (2022) [42] **	Randomized controlled trial	50	1.32 ± 0.68	0.21 ± 0.17	Chandelier-assisted scleral buckling (CSB) and standard SB	102.48 ± 43.76	18(78.3%)	- ERM (44%)	6 M

* Mean values ^†^ visual acuity values in different scales were converted in logMAR ** In comparative studies, chandelier-assisted scleral buckling versus standard scleral buckling, data from the standard scleral buckling group were not included to unify the table. BCVA: best corrected visual acuity; NA: not available SB: scleral buckling; rRD: recurrent retinal detachment; GP: globe perforation; WAVS: wide-angle viewing systems; PPV: pars plana vitrectomy; CSB: chandelier-assisted scleral buckling; IOP: intraocular pressure; CH: choroidal hemorrhage; ERM: epiretinal membrane; SH: subretinal hemorrhage; PVR: proliferative vitreoretinopathy, VH: vitreous hemorrhage, MH: macular hole; SRF: subretinal fluid.

## Data Availability

Publicly available datasets were analyzed in this study. This data can be found here, URL:https://pubmed.ncbi.nlm.nih.gov/ or https://scholar.google.com/ (accessed on 10 March 2023) (see references for DOI).
